# The psychosocial health, experiences and needs of older adults and care partners during the first surge of the COVID-19 pandemic: a mixed-methods study

**DOI:** 10.1186/s12877-022-03427-3

**Published:** 2022-09-15

**Authors:** Allison Marziliano, Edith Burns, Taline Pampanini, Jennifer Tom, Suzanne Ardito, Anum Ilyas, Maria T. Carney, Michael A. Diefenbach, Alex Makhnevich, Liron Sinvani

**Affiliations:** 1 Institute for Health Systems Science, Feinstein Institutes for Medical Research, Northwell Health, 600 Community Drive, Suite 403, Manhasset, NY 11030 USA; 2 Department of Medicine at the Donald and Barbara Zucker School of Medicine at Hofstra, Northwell, 500 Hofstra Boulevard, Hempstead, NY 11549 USA

**Keywords:** Older adult, Care partner, AD/ADRD, COVID-19, Psychosocial health

## Abstract

**Background:**

Minimal research has leveraged qualitative data methods to gain a better understanding of the experiences and needs of older adults (OAs) and care partners of OAs with and without Alzheimer’s Disease (AD) and AD-related dementias (AD/ADRD) during the first surge of the COVID-19 pandemic. In this study, we: 1) quantitatively evaluated the psychosocial health of community-dwelling OAs; 2) quantitatively evaluated the perceived stress of care partners for OAs; 3) qualitatively characterized the experiences and needs of community-dwelling OAs and their care partners; and 4) explored differences in the experiences of care partners of OAs with and without AD/ADRD during the first surge of the COVID-19 pandemic in the New York metropolitan area.

**Methods:**

In this mixed-methods study, telephone interviews were conducted with 26 OAs and 29 care partners (16 of whom cared for OAs with AD/ADRD) from April to July 2020. Quantitative data included: demographics; clinical characteristics (Katz Index of independence in activities of daily living (Katz ADL) and the Lawton-Brody instrumental activities of daily living scale (Lawton-Brody)); and psychosocial health: stress was assessed via the Perceived Stress Scale (PSS), social isolation via the Lubben Social Network Scale (LSNS), loneliness via the DeJong Loneliness Scale (DeJong), and depression and anxiety via the Patient Health Questionnaire-Anxiety and Depression (PHQ). Qualitative questions focused on uncovering the experiences and needs of OAs and their care partners.

**Results:**

OAs (*N* = 26) were mostly female (57.7%), and White (76.9%), average age of 81.42 years. While OAs were independent (M = 5.60, Katz ADL) and highly functional (M = 6.92, Lawton-Brody), and expressed low levels of loneliness, stress, depression and anxiety (M = 1.95 on DeJong; M = 12.67 on PSS; M = 1.05 on PHQ depression; and M = 1.09 on PHQ anxiety), open-ended questions elicited themes of fear and worry. Care partners (*N* = 29) were mostly female (75.9%), White (72.4%), and married (72.4%), and reported moderate stress (M = 16.52 on the PSS), as well as a psychological impact of the pandemic.

**Conclusions:**

Early in the pandemic, OAs reported minimal stress and loneliness; this may have been related to their reports of frequent interaction with family, even if only virtually. By contrast, care partners were moderately stressed and worried, potentially more than usual due to the additional challenges they face when trying to meet their loved ones’ needs during a pandemic.

## Introduction

With the onset of COVID-19, people around the world experienced a dramatic shift in their daily activities. Efforts to stop the spread of illness led to pandemic-related restrictions on in-person contact, including quarantine and shelter-at-home recommendations, which often resulted in unintended consequences, particularly for older adults [1] (OAs, ages 65+). These consequences included difficulty obtaining preferred types of food, reduction in cognitive stimulation, withdrawal of support to help with activities of daily living (ADLs), fear of entering medical facilities to obtain routine care, and reduced likelihood of family/friends visiting [2, 3], all of which have the potential to adversely affect psychosocial health. Psychosocial health is a multidimensional construct encompassing the psychological (depression, anxiety, stress) and social (social isolation, loneliness) components of health. Indeed, there have been multiple studies indicating a negative impact of COVID-19 and related restrictions on OAs’ psychosocial health [4–7]. One study [6] conducted during the pandemic indicated that more than half (54%) of the participants reported worsened loneliness due to COVID-19 that was associated with worsened depression and anxiety. This is concerning, as poor-quality psychosocial health is associated with a range of negative outcomes including obesity [8], lack of exercise [9], and engaging in unhealthy behaviors such as gambling and substance use [10].

There are several gaps in this literature. First, many of the existing studies examining the impact on psychosocial health of pandemic-related social restrictions have been large-scale surveys that rely heavily on quantitative measures [11]; fewer studies have utilized qualitative measures (i.e., open-ended questions) to uncover nuanced experiences and needs of OAs. Second, care partners, non-paid family members or friends who provide assistance to loved ones [12], are less often included as study participants, representing an opportunity to add to the patient’s perspective. Third, this omission means that the unique experience of care partners of OAs with Alzheimer’s Disease (AD) and AD-related dementias (AD/ADRD) has also been minimally explored in this context.

In this mixed-methods study, we: 1) quantitatively evaluated the psychosocial health (perceived stress, social isolation, loneliness, depression, and anxiety) of community-dwelling OAs; 2) quantitatively evaluated the perceived stress of care partners for OAs; 3) qualitatively characterized the experiences and needs of community-dwelling OAs and their care partners; and 4) explored differences, if any, in the experiences of care partners of OAs with and without AD/ADRD, during the first surge of the COVID-19 pandemic in New York, a major metropolitan area of the United States. Of note, this study was conducted in New York at a time when New York was the epicenter of the pandemic.

## Method

### Study procedures

This study was approved by the Institutional Review Board of Northwell Health and the COVID-19 Research Consortium of the affiliated academic medical center. All interviews were conducted between April 14 and July 22, 2020, which corresponds to the peak and decline of the first surge of the pandemic in the New York metropolitan area. To be eligible, patients had to meet the following criteria: (1) aged 65+ years; (2) seen at the academic geriatric faculty outpatient practice within the last 12 months of study start date; (3) able to access a telephone; and (4) able to speak English. To be eligible to participate as a care partner, individuals had to self-identify as a care partner of an OA, defined as those who assist with instrumental activities of daily living (IADLs, such as managing finances and medication, food preparation, housekeeping, laundry, etc.), and ADLs (e.g., eating, bathing, mobility, etc.).

A research assistant (RA) pre-screened the electronic health record of OA patients coming into the clinic and contacted eligible patients and their care partners via telephone. As care partners were asked to speak more generally about their role, we did not require permission from the OA to speak to their care partner. Interested participants provided informed consent and completed a telephone interview. The process to recruit care partners of persons with AD/ADRD was slightly different. For care partners of persons with AD/ADRD, the care partner was contacted directly and invited to participate; no data was collected from the OA patient with AD/ADRD. Of note, our RA deliberately sought out and recruited care partners of persons with AD/ADRD to meet a pre-specified quota for this group.

### Quantitative measures

For patients, the interview consisted of quantitative assessments of demographics and clinical characteristics (i.e., ADLs and IADLs), as well as psychosocial health (perceived stress, social isolation, loneliness, anxiety and depression). For the care partner, the interview consisted of quantitative assessments of demographics and perceived stress (a facet of psychosocial health).

#### Demographics

We collected OAs’ age, gender (male or female) and race (White, Black/African American, Asian, prefer not to say). We collected care partners’ age, gender (male or female), race (White, Black/African American, Asian, prefer not to say), marital status (married/domestic partnership, single/never married or divorced), relationship to the OA (child, spouse/life partner, private aide, or son-in-law), and whether the OA they care for has AD/ADRD (yes or no).

#### Clinical: ADLs

Level of independence in ADLs was evaluated with the Katz Index of independence in activities of daily living [13–17] (Katz ADL), which ranges from 0 (severe functional impairment) to 6 (independently functioning).

#### Clinical: IADLs

IADLs were evaluated with the Lawton-Brody instrumental activities of daily living scale [17, 18] (Lawton-Brody IADL), an instrument measuring independent living skills among 8 domains of functioning. Summary scores for all participants range from 0 (low functioning, dependent) to 8 (high functioning, independent).

#### Psychosocial Health: perceived stress

Perceived stress was evaluated with the Perceived Stress Scale [19] (PSS), the most widely used psychological instrument for measuring the perception of stress in one’s life. Each item is rated on a 5-point Likert scale ranging from 0 (never) to 4 (very often) for 10 items. High scores indicate greater levels of stress.

#### Psychosocial Health: social isolation

Social isolation was evaluated with the Lubben Social Network Scale (LSNS), a 6-item self-report measure of social engagement including family and friends [20]. Response options range from “none” to “nine or more”. The scale correlates with mortality, all case hospitalization, health behaviors, depressive symptoms, and overall physical health.

#### Psychosocial Health: loneliness

Loneliness was measured with the 6-item DeJong Gierveld Loneliness Scale, an evaluation of overall, social and emotional loneliness [21]. Scores range from 0 (least lonely) to 6 (most lonely).

#### Psychosocial Health: anxiety and depression

Anxiety and depression were assessed with the Patient Health Questionnaire-4 [22] (PHQ-4), a 4-item questionnaire answered on a 4-point Likert scale ranging from “not at all” to “nearly every day”.

### Qualitative interviews: experiences and needs

OAs and care partners were asked open-ended questions further evaluating their experiences and needs during the COVID-19 pandemic. The study team, which included a multidisciplinary team of healthcare professionals (geriatricians, hospitalists, behavioral psychologists, etc.) and experienced mixed-methods researchers, created these questions based on their preliminary conversations with OA patients and their care partners in the early days of the pandemic. The study team met several times to refine the questions prior to study implementation.

For older adults, these questions included: “experience: tell me about your usual day now that you are in isolation,” “experience: what precautions do you take when leaving the home?,” and “needs: Is there anything you can think of that would make it easier on older adults to obtain the care or companionship they need when isolating at home?”. For care partners, questions included: “experience: how do you continue providing care while maintaining a safe distance?,” “experience: in what ways has this pandemic affected your ability to provide care?” and “needs: Is there anything you can think of that would make it easier on the older adult to obtain the care or companionship he/she needs when isolating at home?”. See Appendices A and B for the patient and care partner interview guides, respectively.

### Statistical analyses

#### Quantitative analyses

For the quantitative data, overall scores for each of the standardized measures were computed. For categorical measures or items, frequencies and percentages were calculated. For continuous items or measures, means and standard deviations were calculated.

#### Qualitative analyses

For the qualitative data, interviews were audio-recorded and transcribed. We utilized a top-down provisional coding approach to qualitative data analysis [23], in which members of the research team (AM, LS, EB, MD, MC, AM) reviewed the transcriptions with pre-established response themes in mind from prior to the interviews, and created a preliminary codebook of themes in response to each open-ended question. Two coders (TP and JT) reviewed the transcripts independently and coded the absence or presence of each theme for each OA and care partner. The two coders met to discuss agreement. Disagreements over the presence/absence of a theme were settled by a third party (AM).

Quantitative and select qualitative data relevant to being a care partner of an OA with AD/ADRD were also summarized across care partners of OAs with and without AD/ADRD.

## Results

### Overall sample

A total of 26 OAs and 29 care partners of OAs completed telephone interviews. Of the 26 OAs, 24 did not have a care partner enrolled in the study; there were 2 patient/care partner dyads. On average, OAs were 81.42 (SD = 7.47) years old and care partners were 59.62 (SD = 10.30) years old. More than half of OAs were female (57.7%) and over three-quarters were White (76.9%). Of the care partners, three quarters were female (75.9%), and most were White (72.4%), married or in a domestic partnership (72.4%), and were the child of the OA (82.8%). Just over half of the care partners cared for an OA with AD/ADRD (55.2%) See Table 1.

**Table 1:** Demographics

	***N*** = 26 older adults	***N*** = 29 care partners
Variable	M(SD) or n(%)	M(SD) or n(%)
**Age**	81.42 (7.47)	59.62 (10.30)
**Gender**		
Male	11 (42.3)	7 (24.1)
Female	15 (57.7)	22 (75.9)
**Race**		
White	20 (76.9)	21 (72.4)
Black/African American	6 (23.1)	6 (20.7)
Asian	0	1 (3.4)
Prefer not to say	0	1 (3.4)
**Marital Status**		
Married/domestic partnership	–	21 (72.4)
Single/never married	–	5 (17.2)
Divorced	–	3 (10.3)
**Relationship to the older adult**		
Child	–	24 (82.8)
Spouse/life partner	–	2 (6.9)
Private aide	–	2 (6.9)
Son-in-law	–	1 (3.4)
**Does the person you care for have AD/ADRD?**		
Yes	–	16 (55.2)
No	–	13 (44.8)

### Quantitative data: clinical characteristics and psychosocial Health

This sample of OAs was independent (Katz ADL: M = 5.60, SD = 0.87) and highly functional (Lawton-Brody IADL: M = 6.92, SD = 1.78), and reported low levels of stress (PSS: M = 12.67, SD = 7.70), loneliness (DeJong: M = 1.95; SD = 1.53), anxiety (PHQ-anxiety: M = 1.09, SD = 1.57), and depression (PHQ-depression: M = 1.05, SD = 1.33). OAs were moderately socially engaged (LSNS: M = 18.56, SD = 5.96). By contrast, care partners reported moderate levels of stress (PSS: M = 16.52, SD = 5.32). See Table 2.

**Table 2:** Quantitative clinical and psychosocial data

Variable	Older Adult M(SD) Varying N	Care partner M(SD) ***N*** = 29	Possible Range	Interpretation
**Katz ADL (*n*** **= 25)**	5.60 (0.87)	–	0–6	Nearly independent
**Lawton-Brody IADL (n = 25)**	6.92 (1.78)	–	0–8	High functioning
**PSS (*n*** **= 15)**	12.67 (7.70)	16.52 (5.32)	0–40	Low stress, Moderate stress
**LSNS (n = 25)**	18.56 (5.96)	–	0–30	Moderate social engagement
**DeJong (*n*** **= 22)**	1.95 (1.53)	–	0–6	Low loneliness
**PHQ-depression** **(*n*** **= 22)**	1.05 (1.33)	–	0–6	Low depression
**PHQ-anxiety (*n*** **= 22)**	1.09 (1.57)	–	0–6	Low anxiety

*Katz ADL* Katz Index of Independence in Activities of Daily Living

*Lawton-Brody IADL* Lawton-Brody Instrumental Activities of Daily Living Scale

*PSS* Perceived Stress Scale

*LSNS* Lubben Social Network Scale

*DeJong* De Jong Gierveld Loneliness Scale

*PHQ* Patient Health Questionnaire

### Qualitative data: inter-rater agreement

Initially, for the OAs’ qualitative data, the two coders were in agreement 94.3% (2919/3094) of the time; however, after discussion, they reached 100% consensus. For the care partners’ qualitative data, the two coders were in initial agreement 93.9% (5036/5365) of the time; however, after discussion, they reached 100% consensus.

### Older adults’ qualitative data: experiences and needs

Open-ended questions spanned a wide range of topics related to the experiences and needs of OAs during the COVID-19 pandemic. For example, when asked what they did during a usual day sheltering at home, nearly all (96.15%) engaged in screen time (watching TV, iPad, tablet), most (65.38%) engaged in cognitive activity (reading a book, doing crossword puzzles or word searchers), more than half (57.69%) engaged in physical activity (exercise, dancing), and less than half (46.15%) participated in passive activity (listening to music, watching people walk by). When asked what they are doing to stay healthy and keep their spirits up, 100% of those OAs interviewed stated they engaged in self-care related to COVID-19 (i.e., staying home, wearing masks, limiting any activities outside of the home, or wearing protective personal equipment when leaving the house). OAs relied on a number of resources to help meet their needs, including telephone check-in calls (84.62%), contactless delivery (53.85%), and assistance from family, friends or neighbors (46.15%). See Table 3.

**Table 3:** Results from coding of older adults’ qualitative data: experiences and needs (*N* = 26)

Question	Coding of Responses, n(%)
From where do you get news about COVID?	TV/radio/news, 25 (96.15) Political figures/government, 7 (26.92) Social circle/word of mouth, 2 (7.69) Social media, 1 (3.85) Medical professionals, 1 (3.85) Facility announcements, 1 (3.85)
When did you decide it was necessary to shelter in place at home?	When it started, 21 (80.77) When heard older adults were more at risk, 9 (34.62) When heard it was contagious, 2 (7.69)
Who did you make the decision to stay home with?	Alone or with a spouse, 20 (76.92) Family/friends, 5 (19.23) Together as a household, 1 (3.85)
Why did you make the decision to stay home?	Common sense, 9 (34.62) Government said we have to, 3 (11.54) Advertised by TV and newspaper, 3 (11.54) Family/friends encouraged it, 2 (7.69) Facility required it, 1 (3.85)
What do you do during a usual day in isolation?	Screen time, 25 (96.15) Cognitive activity, 17 (65.38) Taking supplements/vitamins, 17 (65.38) Physical activity, 15 (57.69) Interacting with people/pets in person, 14 (53.85) Interacting with people/pets remotely, 14 (53.85) Passive activity, 12 (46.15) Taking care of house, 9 (34.62) Sleeping, 3 (11.54) Going for Drives, 3 (11.54) Eating, 2 (7.69)
How are you feeling?	Scared, 6 (23.08) Nervous, 5 (19.23) Depressed, 4 (15.38) Worrisome, 4 (15.38) Anxious, 4 (15.38) Frustrated, 2 (7.69) Lonely, 1 (3.85)
Why was it necessary for you to leave your home?	Food, 13 (50.0) Medical Care, 9 (34.62) Medications, 6 (23.08) Gas, 2 (7.69) Banking, 1 (3.85)
What precautions did you take when leaving the home?	PPE, 22 (84.62) Social distancing, 14 (53.85) Personal hygiene, 12 (46.15) Product hygiene, 9 (34.62)
Who is coming to your home to help with basic necessities?	Nobody, 10 (38.46) Family, 8 (30.77) Cleaning lady, 4 (15.38) Formal assistance, 3 (11.54) Neighbors/friends, 1 (3.85) Medical help, 1 (3.85)
Who stopped coming into your home because of COVID?	Cleaning lady, 1 (3.85)
What precautions do people take when coming into the home?	PPE, 8 (30.77) Social distancing, 5 (19.23) Lack of precautions, 1 (3.85)
What resources are available to help with your needs?	Telephone check-ins, 22 (84.62) Contactless delivery, 14 (53.85) Family, friends, neighbors helping, 12 (46.15) Online shopping, 6 (23.08) Contactless pick up of needs, 3 (11.54) Telehealth, 3 (11.54) Financial/food stamps, 3 (11.54) Medical house calls, 2 (7.69)
How are you keeping in touch with loved ones while sheltering at home?	Telephone calls, 25 (96.15) Videochatting, 9 (34.62) Socially-distanced visits, 8 (30.77) Email/text-message, 7 (26.92) Posting on social media, 1 (3.85)
Have you or anyone you know come in contact with a COVID positive person?	Yes, personal experience, 12 (46.15) No, 11 (42.31) Yes myself, 1 (3.85)
Has anyone you know died from COVID?	Yes, less personal relationship, 2 (7.69) Yes, more personal relationship, 2 (7.69)
What are you doing to keep yourself safe and healthy and your spirits up?	Self-care, 26 (100) Watching TV, 25 (96.15) Daily supplements/vitamins, 18 (69.23) Cognitive activity, 17 (65.38) Physical activity, 14 (53.85) Social interactions in person, 12 (46.15) Virtual social visits, 8 (30.77) Rituals/routines, 7 (26.92) Hobbies, 6 (23.08) Unfinished business, 6 (23.08) Driving around, 3 (11.54) Social media, 3 (11.54) Distraction/avoidance, 3 (11.54) Sleeping, 2 (7.69) Listening to music, 2 (7.69) Life projects, 1 (3.85) Dancing, 1 (3.85)
Is there anything you can think of that would make it easier on older adults to obtain the care or companionship they need when isolating at home?	Socialization with family, 8 (30.77) Someone to provide needs right to house, 6 (23.08) Safe outdoor space, 2 (7.69) Socialization with community/church, 1 (3.85) Technology education, 1 (3.85) Third party well-being check, 1 (3.85) Financial assistance, 1 (3.85) Physicians coming to the house, 1 (3.85)

### Care partners’ qualitative data: experiences and needs

Data from our semi-structured interviews with care partners revealed that, when asked in what ways the pandemic was affecting their ability to provide care for their loved one, the most frequent response was the need to use increased vigilance (58.62%, giving examples such as handwashing, mask-wearing, monitoring own symptoms). When asked what concerns they had while caring for a loved one during the pandemic, care partners’ most frequently cited keeping the patient safe from contracting COVID-19 (44.83%). When asked how COVID was affecting them personally, care partners most frequent report was experiencing a psychological (55.17%) impact of the pandemic. See Table 4.

**Table 4:** Results from coding of care partners’ qualitative data: experiences and needs (*N* = 29)

Question	Coding of responses, n(%)
Who lives in your loved one’s home with them?	Alone with family nearby, 13 (44.83) Other family, 8 (27.59) Significant other, 7 (24.14) Assisted/Independent Living, 7 (24.14) Aide/Team (part-time), 5 (17.24) Aide/Team (full-time), 4 (13.79)
How do you continue providing care while maintaining a safe distance?	Using PPE, 12 (41.38) Social distancing, 10 (34.48) Not applicable (i.e. care partner lives with patient), 9 (31.03) Increased telephone communication, 5 (17.24) Test ourselves often, 1 (3.45)
How often do you visit?	Lives with older adult, 10 (34.48) Not at all, 7 (24.14) Every day, 5 (17.24) Bi-weekly, 3 (10.34) Every other day, 1 (3.45) Monthly, 1 (3.45) Weekly, 1 (3.45)
What precautions are you taking to stay safe?	Using PPE, 27 (93.10) Social distancing, 19 (65.52) Personal hygiene, 11 (37.93) Isolating at home, 11 (37.93) Product hygiene, 7 (24.14) Self-monitoring, 3 (10.34)
From which sources do you get your news about the coronavirus?	TV/radio/news station, 28 (96.55) Political figures, 6 (20.69) Through work, 5 (17.24) Social media, 3 (10.34) Medical Professions, 2 (6.90) Residential facility/Staff announcement, 1 (3.45)
Do you know anyone who has contracted the coronavirus?	Close friends or family, 12 (41.38) Do not know anyone, 11 (37.93) Someone with less personal experience, 10 (34.48) Knew multiple people with coronavirus, 5 (17.24) Self, 1 (3.45)
Do you know anyone who has died from the coronavirus?	Yes, someone I didn’t know well, 6 (20.69) Yes, close family or friend, 3 (10.34)
As the care partner for an OA, in what ways has this pandemic been affecting your ability to provide care for your loved one?	Increased vigilance, 17 (58.62) Difficulty or inability to visit, 13 (44.83) Changes in ability to provide care, 12 (41.38) Increased emotional distress providing care, 6 (20.69) No change, 6 (20.69) Interference with medical care, 5 (17.24)
Do you think it is necessary to keep a safe distance away?	Absolutely necessary, 21 (72.41) It is respectful to stay away, 2 (6.90) Not necessary because we are all being safe, 1 (3.45)
From your perspective, does the OA understand the current situation with regard to COVID?	Does not full understand due to AD/ADRD, 10 (34.48) Very good understanding, 4 (13.79) Does not fully understand, 3 (10.34) Grasps concept, 2 (6.90)
Has the OA been compliant with social distancing and, if so, how long have they been isolating at home?	OA has no choice but to be compliant, 16 (55.17) Very compliant, 14 (48.28) As much as the OA can remember to comply, 2 (6.90) Increasing compliance as death rate increases, 1 (3.45)
Does he/she remember why they are socially distancing?	Yes, 4 (13.79) No, 2 (6.90)
Have you noticed any changes in the patient in regard to her state of mind, fear, sadness and anxiety?	No effect, 14 (48.28) Anxious, 6 (20.69) Scared, 5 (17.24) Confused/forgetful, 3 (10.34) Unhappy, 3 (10.34) Angry, 2 (6.90) Nervous, 1 (3.45) Regressing, 1 (3.45)
What sort of tasks do you help the OA with?	IADLs, 23 (79.31) ADLs, 10 (34.48) OA is independent, 3 (10.34)
How has that been affected the past few weeks?	Decreased ability to help OA, 7 (24.14)
As their care partner, how are necessities such as food and medication being provided?	Family member brings it to them, 23 (79.31) Delivery of purchased food/medications, 14 (48.28) Provided by facility, 8 (27.59) Aide, 6 (20.69) Food program, 2 (6.90)
What items have been difficult to obtain?	Medications, 5 (17.24) Paper goods/toiletries, linens, 3 (10.34) Food, 2 (6.90) PPE, 1 (3.45)
Why has it been difficult to obtain these items?	Long lines, 3 (10.34) Lack of supplies, 3 (10.34)
Have you used any additional resources to assist with these needs?	Online purchases, 14 (48.28) Help from others (self, family, neighbors), 5 (17.24) Virtual medical consultations, 4 (13.79) Delivery of already-prepared food, 4 (13.79) Virtual technology for socialization, 3 (10.34) Picture slideshow, 1 (3.45)
What have you found to be the most challenging aspect of providing care for your loved one during this pandemic?	Emotional support, 7 (24.14) Obtaining medical supplies, 3 (10.34) Physical care, 2 (6.90) Obtaining food, 1 (3.45)
Why?	Safety guidelines, 4 (13.79) Out of stock, 2 (6.90) Fear, 2 (6.90) Long lines, 1 (3.45)
What concerns do you personally have in caring for an OA in this pandemic?	Keeping the patient safe, 13 (44.83) Social isolation of the OA, 9 (31.03) Keeping oneself safe, 7 (24.14) Functional/cognitive decline of OA, 4 (13.79) Meeting health needs of the OA, 4 (13.79) No concerns, 4 (13.79) Meeting physical needs of the OA, 2 (6.90) Meeting material needs of the OA, 1 (3.45) Financial problems, 1 (3.45)
From your perspective, how is your OA coping with the current situation?	Well or very well, 9 (31.03) Anxious, 6 (20.69) Emotional distress, 5 (17.24) Frustrated, 3 (10.34) Lonely, 2 (6.90) Angry, 2 (6.90) Sad/depressed/tearful, 2 (6.90)
In what ways is the pandemic affecting your loved one?	No change, 10 (34.48) Worsening cognition, 2 (6.90) Positively, 1 (3.45)
What activities is the OA doing to stay healthy and occupied while at home?	Self-care, 28 (96.55) Watch TV, 22 (75.86) Cognitive Activity, 12 (41.38) Physical Activity, 9 (31.03) Social interactions in person, 8 (27.59) Distraction/keeping busy, 6 (20.69) Virtual social visits, 5 (17.24) Passive Activity, 4 (13.79) Rituals/routines, 4 (13.79) Hobbies, 3 (10.34) Daily supplements/vitamins, 3 (10.34) Unfinished business, 2 (6.90) Sleeping, 2 (6.90) Eating healthy, 1 (3.45) Dancing, 1 (3.45)
How are you as the care partner staying safe and healthy during this time?	Self-care, 29 (100) Watching TV, 25 (86.21) Cognitive activity, 13 (44.83) Physical activity, 11 (37.93) Social interactions in person, 10 (34.48) Virtual social visits, 10 (34.48) Work from home, 8 (27.59) Social media, 5 (17.24) Distraction/Keeping busy, 4 (13.79) Homeschooling, 3 (10.34) Rituals/routines, 2 (6.90) Hobbies, 2 (6.90) Eating healthy, 2 (6.90) Music, 2 (6.90) Daily supplements/vitamins, 2 (6.90) Unfinished business, 1 (3.45)
In what ways would you say COVID is affecting you?	Psychologically, 16 (55.17) Socially/not able to go out, 9 (31.03) Financially, 4 (13.79) Employment change, 4 (13.79) Less time, 3 (10.34) Physically/changes in sleep, 1 (3.45) More time, 1 (3.45) Increased appreciation, 1 (3.45) Increased ambiguity, 1 (3.45) None, 1 (3.45)
Who can you lean on for support during this time?	Immediate family, 23 (79.31) Friends/neighbors, 17 (58.62) Distant family, 8 (27.59) Other aides, 1 (3.45)
Does the patient have anyone he/she keeps in touch with outside of the home?	Immediate family, 16 (55.17) Friends/neighbors, 2 (6.90) Religion/spiritual affiliation, 1 (3.45) Distant family, 1 (3.45)
Is there anything you can think of that would make it easier on the OA to obtain the care or companionship he/she needs when isolating at home?	Third-party well-being checks, 7 (24.14) Virtual capabilities or training, 6 (20.69) Socialization for the community/church, 5 (17.24) Someone to provide needs right to the house, 3 (10.34) More virtual engagement with healthcare experts, 2 (6.90) Keeping a routine for the OA, 1 (3.45)

### Comparing care partners of OAs with and without AD/ADRD

Additionally, we explored differences in the profiles and experiences of care partners of OAs with and without AD/ADRD. Demographics (age, gender, race, marital status, relationship to the OA) were comparable between care partners of OAs with and without AD/ADRD. Specifically, the demographics for care partners of OAs with AD/ADRD were mostly female (75%), White (75%), married or in a domestic partnership (75%), and the child of the OA (87.5%), with a mean age of 58.38 (SD = 8.29) years. The demographics for care partners of OAs without AD/ADRD were, similarly, mostly female (76.92%), White (69.23%), married or in a domestic partnership (69.23%), and the child of the OA (76.92%), with a mean age of 61.15 (SD = 12.54) years. See Table 5.

**Table 5:** Exploring differences between care partners of OAs with and without AD/ADRD

	Care partners of OAs with AD/ADRD (***n*** = 16)	Care partners of OAs without AD/ADRD (***n*** = 13)
**Variable**	**M(SD) or n(%)**	**M(SD) or n(%)**
**Age**	58.38(8.29)	61.15(12.54)
**Gender**		
Male	4 (25.0%)	3 (23.08%)
Female	12 (75.0%)	10 (76.92%)
**Race**		
White	12 (75.0%)	9 (69.23%)
Black/African American	4 (25.0%)	2 (15.38%)
Asian	0 (0%)	1 (7.69%)
Prefer not to say	0 (0%)	1 (7.69%)
**Marital status**		
Married/domestic partnership	12 (75.0%)	9 (69.23%)
Single/never married	3 (18.75%)	2 (15.38%)
Divorced	1 (6.25%)	2 (15.38%)
**Relationship to OA**		
Child	14 (87.5%)	10 (76.92%)
Spouse/life partner	1 (6.25%)	1 (7.69%)
Private Aide	1 (6.25%)	1 (7.69%)
Son-in-law	0 (0%)	1 (7.69%)
**Perceived Stress**	15.69(5.49)	17.54(5.13)
**Who lives in your loved one’s home with them?**		
Alone with family nearby	8 (50.0%)	5 (38.46%)
Other family	3 (18.75%)	5 (38.46%)
Significant other	4 (25.0%)	3 (23.08%)
Assisted/independent living	3 (18.75%)	4 (30.77%)
Aide/Team (part time)	4 (25%)	1 (7.69%)
Aide/Team (full time)	4 (25%)	0 (0%)
**How often do you visit?**		
Lives with older adult	4 (25%)	6 (46.15%)
Not at all	3 (18.75%)	4 (30.77%)
Every day	5 (31.25%)	0 (0%)
Bi-weekly	2 (12.5%)	1 (7.69%)
Every other day	0 (0%)	1 (7.69%)
Monthly	0 (0%)	1 (7.69%)
Weekly	1 (6.25%)	0 (0%)
**Does the OA understand the current situation with regard to COVID?**		
Does not fully understand	11 (68.75%)	2 (15.38%)
Very good understanding	3 (18.75%)	1 (7.69%)
Grasps concept	2 (12.5%)	0 (0%)
**Has the OA been compliant with social distancing?**		
Yes, has no choice	11 (68.75%)	5 (38.46%)
Very compliant	6 (37.5%)	8 (61.54%)
As much as he/she can remember	1 (6.25%)	1 (7.69%)
Increasing compliance as death rate increases	1 (6.25%)	0 (0%)
**Have you noticed any changes in the patient with regard to state of mind, fear, sadness and anxiety?**		
No effect	11 (68.75%)	3 (23.08%)
Anxious	2 (12.5%)	4 (30.77%)
Scared	2 (12.5%)	3 (23.08%)
Confused/forgetful	2 (12.5%)	1 (7.69%)
Unhappy	0 (0%)	3 (23.08%)
Angry	1 (6.25%)	1 (7.69%)
Nervous	1 (6.25%)	0 (0%)
Regressing	1 (6.25%)	0 (0%)
**From your perspective, how is the OA coping with the current situation?**		
Well or very well	4 (25%)	5 (38.46%)
Anxious	3 (18.75%)	3 (23.08%)
Emotional distress	2 (12.5%)	3 (23.08%)
Frustrated	1 (6.25%)	2 (15.38%)
Lonely	1 (6.25%)	1 (7.69%)
Angry	1 (6.25%)	1 (7.69%)
Sad/depressed/tearful	0 (0%)	2 (15.38%)
**In what ways is the pandemic affecting your loved one?**		
No change	8 (50.0%)	2 (15.38%)
Worsening cognition	1 (6.25%)	1 (7.69%)
Positively	1 (6.25%)	0 (0%)

Care partners of OAs without AD/ADRD reported higher levels of stress than care partners of OAs with AD/ADRD. When examining the qualitative data, we found that compared to those care partners of OAs without AD/ADRD, care partners of OAs with AD/ADRD were more likely to visit every day (0% versus 31.25%), more likely to report that the pandemic has no effect on their loved one (23.08% versus 68.75%), less likely to report that their loved one was unhappy (23.08% versus 0%), and more likely to report no change in their loved one due to the pandemic (15.38% versus 50%). Additionally, based on care partner report, compared to the OAs without AD/ADRD, the OAs with AD/ADRD were more likely to have an aide live in the home (7.69% versus 25%), and less likely to fully understand the current situation with regard to COVID-19 (15.38% versus 68.75%). See Table 5.

## Discussion

Although COVID-19 is an infectious illness, substantial research has and is continuing to focus on the psychosocial impact of the disease and pandemic-related restrictions [1–4]. Our study sought to use quantitative and qualitative methodology to gain a better understanding of the psychosocial health, unique experiences and needs of OAs and care partners during the first surge of the pandemic. We found that this sample of OAs were independent and highly functional (based on ADLs and IADLs). Based on the quantitative measures, OAs expressed low levels of stress, loneliness, depression and anxiety, as well as moderate social engagement. Care partners reported moderate stress, which was also endorsed in qualitative interviews, indicating a substantial group of care partners were psychologically affected by the pandemic. Although the demographics were comparable between care partners of OAs with and without AD/ADRD, our exploratory comparison yielded differences between the two groups, including that care partners of OAs without AD/ADRD reported higher levels of stress than care partners of OAs with AD/ADRD.

The finding that most of the OAs in our sample quantitatively reported low levels of stress, anxiety, depression, and loneliness was somewhat unexpected. In contrast to our findings, there is a great deal of evidence to support a negative impact of the pandemic and related restrictions on OAs’ psychosocial health. A review of 24 papers including individuals of all ages indicated that the negative psychological effects of quarantine include post-traumatic stress symptoms, confusion and anger [4]. Another study found increases from pre-pandemic levels in loneliness, depression and anxiety among OAs [6]. We offer several explanations for our findings. First, it is possible that this small sample represented a resilient sub-group of OAs, given their status as community-dwelling, most having care partners, and their reports of being mostly independent and high functioning in ADLs and IADLs. Findings may be quite different for OAs living in a facility setting or without care partners’ assistance and support. Second, as these interviews were conducted early on in the initial months of the pandemic, it is possible that these OAs had not yet been “worn down” by many months of pandemic-related restrictions, or they were motivated by the hope that restrictions would be lifted soon. Third, it may also be the case that OAs demonstrate a unique resiliency in the face of trauma. Indeed, research has highlighted that, in other experiences such as war, earthquakes, and terrorist attacks, OAs are notably resilient psychologically, and can draw on a lifetime of experience and perspective to meet new challenges and navigate difficult times [24].

It is noteworthy that our qualitative interviews with OAs elicited, albeit minimal, themes of fear, anxiety, and worry, findings that would have gone undetected had we relied solely on quantitative data collection methods. Thus, our study highlights the importance of including qualitative methods as part of behavioral research studies. Quantitative assessment instruments are designed to capture the presence or absence of specific constructs; while they have their strengths, they are unable to provide a deeper understanding of the why and how of phenomena. In our study, the qualitative data plays the critically important role of providing a more detailed picture of OAs and care partners’ experiences and needs during the pandemic.

Our qualitative interview with OAs highlighted that this group engaged in many different strategies to keep connected, busy and occupied while staying safe at home. While some of these activities are “cognitively passive and/or sedentary” (e.g. watching TV, sleeping, going for drives), others include “cognitive activity” such as reading, crossword puzzles, and word games. The use of technology among these OAs was substantial (interacting with people/pets remotely), suggesting that leveraging technology-based interventions for OAs is an important area for future research. Still, our data indicate that the telephone remains the main way for OAs to connect with loved ones and meet their own needs.

Care partners reported moderate levels of stress on both quantitative and qualitative measures, with over half indicating the pandemic was affecting them psychologically. Even pre-COVID-19, care partners of individuals with a variety of illnesses reported high levels of distress and burden [25]. Our qualitative data suggest an additional layer of activities that care partners of OAs shouldered due to COVID-19 and pandemic-related restrictions (e.g. increased vigilance needed when providing care and keeping the OA safe from contracting COVID-19), potentially contributing to their stress levels. Future research should explore the potential to improve both patient and care partners’ psychosocial health via interventions that support the care partners.

Although the demographics were similar, we found differences in the responses of care partners with and without AD/ADRD. Particularly, we found that care partners of cognitively intact OAs reported more stress than care partners of OAs with AD/ADRD. There are several explanations for these findings. First, it is possible that, due to their cognitive deficits, OAs with AD/ADRD were unaware and unable to grasp the severity of the pandemic, making it less likely that they would feel sadness, distress, depressed or lonely- all feelings that would make care partnering more difficult. Second, OAs with AD/ADRD may be less likely to or even unable to counter their care partners’ pleas to social distance, wear masks and stay at home. OAs who are cognitively intact are capable of interpreting requests to stay home as an attack on their autonomy, leading to a more tumultuous care partnering dynamic. Third, it is possible that, compared to other sub-groups of care partners, care partners of OAs with AD/ADRD experienced less of a change in their daily responsibilities as a result of the pandemic. As our sample was small, future research ought to explore these potential differences with larger sample sizes.

### Limitations

Our study has several limitations. First, although our questions were asked in the context of the COVID-19 pandemic, we do not have data from the pre-pandemic period to compare responses. Second, though our sample size is appropriate for qualitative data collection and analysis, its small size prevented us from determining statistically significant differences between sub-groups of OAs or care partners. Third, due to our limited resources with which to conduct this study, we were only able to include English-speakers.

## Conclusions

This study is the first to incorporate a wide range of open-ended questions to shed light on the unique needs and experiences of OAs and their care partners during the first surge of the COVID-19 pandemic. Future studies should focus on examining the psychosocial health of OAs and care partners at later points in the pandemic, and exploring potential interventions to improve OA and care partners’ outcomes through addressing care partners’ stress.

## Data Availability

The datasets generated and/or analyzed during the current study are not publicly available due to that this manuscript reports mostly on qualitative data, in which we analyzed transcriptions of audio-recorded interviews, but are available from the corresponding author on reasonable request.

## Abbreviations

OAs: older adults
AD: Alzheimer’s Disease
AD/ADRD: AD-related dementias
Katz ADL: Katz Independence in Activities of Daily Living
Lawton-Brody IADL: Lawton Brody Instrumental Activities of Daily Living Scale
PSS: Perceived Stress Scale
LSNS: Lubben Social Network Scale
DeJong: DeJong Loneliness Scale
PHQ: Patient Health Questionnaire
ADLs: activities of daily living
IADLs: instrumental activities of daily living
RA: research assistant
